# Selection and Validation of Reference Genes for RT-qPCR Normalization in *Bradysia odoriphaga* (Diptera: Sciaridae) Under Insecticides Stress

**DOI:** 10.3389/fphys.2021.818210

**Published:** 2022-01-11

**Authors:** Haiyan Fu, Tubiao Huang, Cheng Yin, Zhenhua Xu, Chao Li, Chunguang Liu, Tong Wu, Fuqiang Song, Fujuan Feng, Fengshan Yang

**Affiliations:** ^1^Engineering Research Center of Agricultural Microbiology Technology, Ministry of Education, Heilongjiang University, Harbin, China; ^2^Heilongjiang Provincial Key Laboratory of Ecological Restoration and Resource Utilization for Cold Region, School of Life Sciences, Heilongjiang University, Harbin, China; ^3^College of Life Science, Northeast Forestry University, Harbin, China

**Keywords:** *Bradysia odoriphaga*, RT-qPCR, reference gene, insecticides stress, normalization

## Abstract

*Bradysia odoriphaga* (Diptera: Sciaridae) is the most serious root maggot pest which causes substantial damage to the Chinese chive. Organophosphate (OP) and neonicotinoid insecticides are widely used chemical pesticides and play important roles in controlling *B. odoriphaga*. However, a strong selection pressure following repeated pesticide applications has led to the development of resistant populations of this insect. To understand the insecticide resistance mechanism in *B. odoriphaga*, gene expression analysis might be required. Appropriate reference gene selection is a critical prerequisite for gene expression studies, as the expression stability of reference genes can be affected by experimental conditions, resulting in biased or erroneous results. The present study shows the expression profile of nine commonly used reference genes [*elongation factor 1α (EF-1α)*, *actin2 (ACT)*, *elongation factor 2α (EF-2α)*, *glucose-6-phosphate dehydrogenase (G6PDH)*, *glyceraldehyde-3-phosphate dehydrogenase (GAPDH)*, *ribosomal protein L10 (RPL10)*, *ribosomal protein S3 (RPS3)*, *ubiquitin-conjugating enzyme (UBC)*, and *α-tubulin (TUB*)] was systematically analyzed under insecticide stress. Moreover, we also evaluated their expression stability in other experimental conditions, including developmental stages, sexes, and tissues. Five programs (NormFinder, geNorm, BestKeeper, RefFinder, and Δ*Ct*) were used to validate the suitability of candidate reference genes. The results revealed that the most appropriate sets of reference genes were *RPL10* and *ACT* across phoxim; *ACT* and *TUB* across chlorpyrifos and chlorfluazuron; *EF1*α and *TUB* across imidacloprid; *EF1*α and *EF2*α across developmental stages; *RPL10* and *TUB* across larvae; *EF1*α and *ACT* across tissues, and *ACT* and *G6PDH* across sex. These results will facilitate the standardization of RT-qPCR and contribute to further research on *B. odoriphaga* gene function under insecticides stress.

## Introduction

*Bradysia odoriphaga* Yang et Zhang (Diptera: Sciaridae) is a serious soil pest in China that feeds on 7 plant families and more than 30 plant species, including Chinese chive (Liliaceae), onion (Liliaceae), Chinese cabbage (Cruciferae), lettuce (Asteraceae), and so on (Li W. X. et al., 2015; Yang Y. T. et al., 2015). The main host plant of *B. odoriphaga* is Chinese chive (*Allium tuberosum* Rottle ex Spreng). Chinese chive is a perennial vegetable with a high economic value and is grown over a vast geographic area from Asia through the Middle East, to Europe and North America, and is widely cultivated in China. *B. odoriphaga* larvae usually gather in the roots, bulbs, and even in immature stems of Chinese chives, making the pest hard to control and allowing it to cause significant production losses of Chinese chives (Zhang et al., 2013; Chen et al., 2018). Yield loss of Chinese chive caused by *B. odoriphaga* has been reported to vary from 40 to 60% (Li et al., 2007). So far, the control efforts against *B. odoriphaga* still largely rely on the application of chemical insecticides, such as organophosphate (OP) and neonicotinoid insecticides (Chen et al., 2017). Phoxim, chlorpyrifos, imidacloprid, and chlorfluazuron are very popular insecticides that are used extensively for the purpose of *B. odoriphaga* control. Unfortunately, *B. odoriphaga* has developed increased resistance to insecticides because of heavy reliance on chemical insecticides (Chen et al., 2017). To investigate insecticide resistance mechanisms and promote integrated pest management (IPM) strategies, researchers have studied several pests over the past few decades and achieved important progress in several areas, including genomics (Xiao et al., 2021), transcriptomics (Cheng et al., 2020; Nazar et al., 2020; Nor Muhammad et al., 2020; Wang et al., 2020; Fu et al., 2021; Zou et al., 2021), proteomics (Prajapati et al., 2020; Chen et al., 2021), insecticide resistance (Wang et al., 2020; Gong et al., 2021; Ullah et al., 2021), RNA interference (Koo et al., 2020; Silver et al., 2021), and gene functions (Yu et al., 2020; Li L. L. et al., 2021; Luo et al., 2021). However, further studies on the mechanism of insecticide resistance are required to clarify the genes directly involved in resistance and regulatory mechanisms associated with those genes.

At present, real-time quantitative PCR is considered a reliable method to determine minor deviations in mRNA expression levels of a target gene due to its speed, accuracy, sensibility, throughput, cost, and reproducibility. The results of RT-qPCR must be normalized using reference genes because the threshold cycle (*Ct*) values are influenced by RNA quality and quantity, primer characteristics, PCR conditions, and variable transcriptional efficiencies. Since the validity and accuracy of RT-qPCR are highly dependent on the reference genes, it is imperative to identify the ideal candidate reference genes. An ideal reference gene should be constitutively and equally expressed in different cell types and tissues, regardless of internal and external factors or physiological cycles (Castanera et al., 2015). Many housekeeping genes that are necessary for regular cell functions have been universally used to normalize gene expression (Adeyinka et al., 2019; Chen et al., 2020). The top 10 most frequently used reference genes are *Actin*, *RPL*, *Tubulin*, *GAPDH*, *RPS*, *18S*, *EF1*α, *TATA*, *HSP*, and *SDHA* (Lü et al., 2018). Several methods and programs have been developed to evaluate the stability of reference genes, including the Δ*Ct* method (Silver et al., 2006), BestKeeper (Pfaffl et al., 2004), NormFinder (Andersen et al., 2004), geNorm (Vandesompele et al., 2002), and a web-based tool RefFinder (Xie et al., 2012). However, accumulating data of reference genes studies showed that an ideal reference gene that can keep stability in various experimental conditions does not exist (Yuan et al., 2014). Therefore, the reference genes should be selected cautiously, and their stability be validated before they are used under specific experimental conditions.

Consideration of the significance and diverse specificity of reference genes, many reference gene sets have been validated in various insect species, such as *Spodoptera frugiperda* (Zhou et al., 2021), *Rhopalosiphum padi* (Li M. et al., 2021), *Aquatica leii* (Fu and Meyer-Rochow, 2021), *Tuta absoluta* (Yan et al., 2021), *Dichelops melacanthus* (Pinheiro et al., 2020), *Thermobia domestica* (Bai et al., 2020), *Apolygus lucorum* (Luo et al., 2020), *Lymantria dispar* (Yin et al., 2020), *Drosophila melanogaster* (Kim et al., 2020), *Phenacoccus solenopsis* (Zheng et al., 2019), *Chilo partellus* (Adeyinka et al., 2019), *Harmonia axyridis* (Yang et al., 2018), *Liriomyza trifolii* (Chang et al., 2017), *Myzus persicae* (Kang et al., 2017), and *B. odoriphaga* (Shi et al., 2016) under various experimental conditions. However, a universal reference gene has not yet been identified. Therefore, the lack of a single universal reference for *B. odoriphaga* is not surprising. In this case, it’s important to choose reliable reference genes for gene expression analysis under various experimental conditions. Though appropriate references genes have been identified in *B. odoriphaga* under different biotic and abiotic conditions (Shi et al., 2016; Tang et al., 2019), a piece of comprehensive information is lacking for *B. odoriphaga* stressed by different groups of insecticides. Therefore, in this study, nine commonly used reference genes *elongation factor 1α (EF-1α)*, *actin2 (ACT)*, *elongation factor 2α (EF-2α)*, *glucose-6-phosphate dehydrogenase (G6PDH)*, *glyceraldehyde-3-phosphate dehydrogenase (GAPDH)*, *ribosomal protein L10 (RPL10)*, *ribosomal protein S3 (RPS3)*, *ubiquitin-conjugating enzyme (UBC)*, and *α-tubulin (TUB)* were analyzed to assess their suitability for normalizing RT-qPCR data for *B. odoriphaga* under the stress of insecticides (phoxim, chlorpyrifos, imidacloprid, and chlorfluazuron). Additionally, the effects of developmental stages, tissues, and sexes were also evaluated. The objective of the present work was to identify different sets of suitable reference genes for further studies of toxicology-related target genes in *B. odoriphaga*.

## Materials and Methods

### Insects

*B. odoriphaga* was originally collected from The Institute of Plant Protection, Academy of Agricultural Sciences, Tianjin, China (39°10′36″N, 117°05′86″E) in 2018. The individuals were reared on scallions in an incubator at 20 ± 1°C, and 65 ± 5% relative humidity with a 12-h light:12-h dark photoperiod in culture dishes (Φ = 90 mm) filled with 2.5% agar solution at the liquid level of 0.5 mm and covered with filter paper.

### Chemicals

Formulated insecticides, 50% phoxim EC, 40% chlorpyrifos EC, 20% imidacloprid SE, and 5% chlorfluazuron SE were manufactured by Xuzhou Shennong Chemical Co., Ltd., JiangSu, China, and kept in a refrigerator.

### Analyzed Factors

The effects of the following factors on candidate reference genes mRNA were measured: insecticides (phoxim, chlorpyrifos, imidacloprid, and chlorfluazuron), developmental stages, tissues, and sexes. The samples processed by each factor were flash-frozen in liquid nitrogen and then stored at −80°C until analyzed by RT-qPCR. Each factor was assessed in four independent experiments.

### Determination of LC_50_ Value of Insecticides

Groups of 15, third instar larvae were sprayed in a culture dish with 600 μL phoxim, chlorpyrifos, imidacloprid or chlorfluazuron, half on the body, half around, and fed scallion stained with pesticide (Li Z. N. et al., 2015). The control group was sprayed with distilled water. The number of dead individuals was checked after 24 h at 20°C and RH: 60–70%. LC_50_ was calculated for all the samples by survival analysis using SPSS 19.0 software for Windows (SPSS Inc., Chicago, IL, United States).

### Insecticides Stress

The treatment groups of third instar larvae were sprayed with the LC_50_ value of phoxim, chlorpyrifos, imidacloprid, or chlorfluazuron. The control group was sprayed with distilled water. After 24, 48, and 72 h, 23 larvae in total were collected, flash-frozen, and stored.

### Developmental Stages

*B. odoriphaga* samples were collected in a dish at each of the six developmental stages: first instar larvae, second instar larvae, third instar larvae, fourth instar larvae, pupa, and adult. Each dish contained 100 samples.

### Tissues

The head, thorax, and abdomen from the fourth instar larvae were dissected by a dissection needle and a tweezer under a stereomicroscope. For each tissue, four replicates of 100 samples were collected.

### Sexes

Hundred male and 100 female wingless *B. odoriphaga* adults were collected, flash-frozen in liquid nitrogen, and stored at −80°C until analyzed by RT-qPCR.

### Primer Design

A set of nine candidate reference genes included *EF1*α, *EF2*α, *ACT*, *GAPDH*, *G6PDH*, *RPL10*, *RPS3*, *TUB*, and *UBC*. All of these genes are commonly used as reference genes in RT-qPCR analysis of other insects (Lü et al., 2018). The sequences of genes were obtained from *B. odoriphaga* transcriptome data (Chen et al., 2019). Primers were designed by NCBI Primer-BLAST^1^. The secondary structure of DNA template was predicted by the mfold web server^2^. Parameters were set as PCR products size 80–200 bp and size of primer 18–25 bp. Primers were synthetized by JINKAIRUI company, Wuhan, China. The details regarding the RT-qPCR primers are provided in Table 1.

**TABLE 1:** Primer sequences and amplicon characteristics of the nine reference genes in *B. odoriphaga* samples.

Gene symbol	Gene name	(Putative)function	Primer sequences (5′ → 3′)	Amplicon length (bp)	*E* (%) *	*R* ^2^ **
*EF1*α	*Elongation factor 1*α	Structural constituent of ribosome	F: TTTTGGCCTTCACCCTTGGT R: AACGGTTCTCGCTGAATGGT	87	108.2	0.996
*ACT*	*Actin2*	Structural constituent of ribosome	F: AGAGCAAACGTGGTATCCTTACTT R: CTGGATGTTCTTCGGGTGCG	132	103.7	0.997
*EF2*α	*Elongation factor 2*α	Involved in cell motility, structure, and integrity	F: CTGCTGCAATCACAGCCAAG R: GGAAAGCTTGACCGCCAGTA	237	102.3	0.996
*G6PDH*	*Glucose-6-phosphate dehydrogenase*		F: ATCACTCATTCGGCGCTCTT R: CGGTACAAGTACCACAGCGT	150	98.8	0.998
*GAPDH*	*Glyceraldehyde-3-phosphate dehydrogenase*	Glycolytic enzyme	F: GGTCGTTTGGTACTTCGTGC R: GACCACCAAGAAGCCACCTT	162	98.7	0.998
*RPL10*	*Ribosomal protein L10*	Structural constituent of ribosome	F: AAGCGTTTCTCCGGAACTGT R: TATGCGGGTAACCAAGAGCG	115	106.4	0.997
*RPS3*	*Ribosomal protein S3*	Structural constituent of ribosome	F: TCTACGCAGAAAAGGTGGCA R: ACGAACGGCTAATCCACCAG	92	101.4	0.998
*UBC*	*Ubiquitin-conjugating enzyme*		F: CTTCTTCAGGAGCCCGTACC R: CTCGAATGGGGAGTCTGACG	102	101.2	0.998
*TUB*	*α-Tubulin*	Cytoskeleton structural protein	F: CACGTGCCGTTTTGGTTGAT R: TTACCGGCACCAGATTGACC	115	100.2	0.999

*Real-time qPCR efficiency (calculated by the standard curve method).

**Regression coefficient calculated from the regression line of the standard curve.

### Total RNA Extraction and cDNA Synthesis

Total RNA was extracted using the TRNzol Universal Reagent as described by the manufacturer (TaKaRa Bio, Dalian, China). The quantity and quality of RNA samples were assessed with a spectrophotometer 2000 (Thermo Scientific, Wilmington, DE, United States). RNA samples with OD ratio (A_260_/A_280_) ranging between 1.9 and 2.12 were selected for reverse transcription. Following the manufacturer’s instructions, the cDNA was synthesized using the Prime script TMRT reagent kit (TaKaRa Bio, Dalian, China). The synthesized cDNA was stored at −20°C.

### RT-qPCR

The PCR reaction system was structured by SYBR Premix Ex Taq II kit (TaKaRa, Dalian, China). Each reaction was operated in a 20-μL solution including 2 μL mixture, 10 μL SYBR Premix Ex Taq II, 0.8 μL forward primer, 0.8 μL reverse primer, and 6.4 μL distilled water. The mixture was cDNA synthesized in different reverse transcription conditions. The amplification conditions for the RT-qPCR were set as following: 95°C for 30 s; followed by 40 cycles of 95°C for 5 s, 60°C for 34 s. The corresponding RT-qPCR efficiencies (*E*) were counted employing the equation: *E* = (10^[–1/slope]^ − 1) × 100, with cDNA gradient dilution (1, 1/5, 1/25, 1/125, 1/625, and 1/3125) set as abscissa and *Ct* value as ordinate (Pfaffl, 2001). All samples were set three biological replicates and three technical replicates. The *Ct* values were obtained by analyzing the result from RT-qPCR using the SDS software of ABI 7500 (version 1.4).

### Data Analysis

Data from RT-qPCR were analyzed by software SDS Shell.exe for ABI7500. The values were given as cycle threshold (*Ct*) numbers. All the *Ct* values were the average means of three biological replicates. The Δ*Ct* method and three analysis applets NormFinder version 0.953^3^, GeNorm version 3.5^4^, and BestKeeper^5^ were used to validate the stability of candidate reference genes. The comprehensive rank and a suitable number of reference genes were calculated by RefFinder^6^ and GeNorm, respectively.

## Results

### Verification of PCR Amplicons and PCR Amplification Efficiencies

**FIGURE 1:**
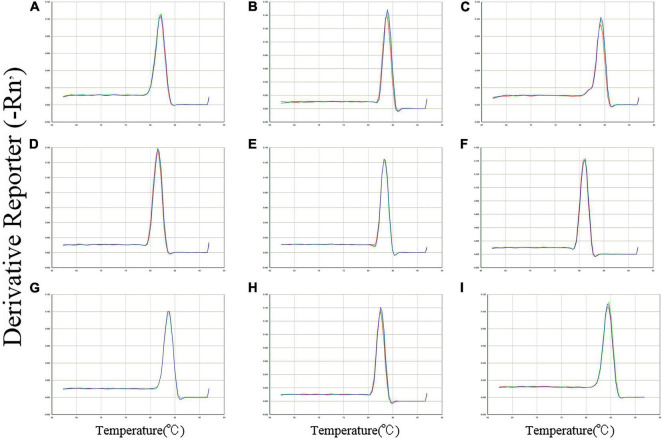
Specificity of primer pairs for RT-qPCR amplification in *B. odoriphaga*. Melting curves with single peaks were produced for all amplicons. **(A)** *EF1*α; **(B)** *EF2*α; **(C)** *ACT*; **(D)** *GAPDH*; **(E)** *G6PDH*; **(F)** *RPL10*; **(G)** *RPS3*; **(H)** *TUB*; and **(I)** *UBC.*

The specific amplification of all primer pairs of candidate reference genes was confirmed with regular PCR and RT-qPCR. The PCR amplifications were identified by sequencing clones of the open reading frame (ORF). The results were consistent with the results of transcriptome sequencing. A single amplification peak for each candidate reference gene was observed in the melting curve (Figure 1). The size of amplicons ranged from 87 to 237 bp. The amplification efficiencies (*E*) for these genes varied from 98.7% for *GAPDH* to 108.2% for *EF1*α, and the correlation coefficients (*R*^2^) varied from 0.999 to 0.996 (Table 1).

### Expression Profiles of Candidate Reference Genes

**FIGURE 2:**
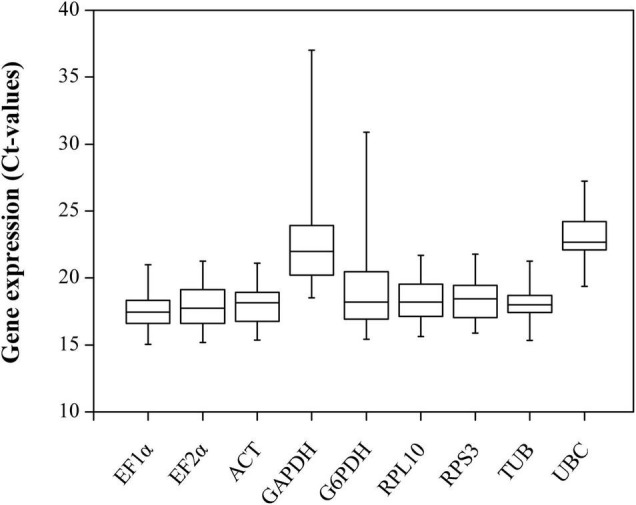
Candidate reference genes expression profiles in *B. odoriphaga.* The expression data are presented as mean *Ct* values for duplicate samples. Whiskers represent the maximum and minimum values. The lower and upper borders of boxes represent the 25th and 75th percentiles, respectively. The line across the box indicates the median *Ct* value.

The raw *Ct* values of the nine candidate reference genes for RT-qPCR were collected and are shown in Figure 2. The *Ct* values varied from 15.02 (*EF-1*α) to 37.26 (*GAPDH*), and the average *Ct* values ranged from 17.30 (*EF-1*α) to 22.10 (*UBC*), which indicates that noticeable differences exist in the expression profiles. Low *Ct* values correspond to high expression levels. Therefore, *EF-1*α exhibited the highest expression abundance, and *UBC* expressed the lowest level. Moreover, *Ct* values have also shown the differential expression variability, and *EF-1*α and *TUB* had a relatively narrow *Ct* range than other genes, indicating that these two genes might be expressed more stably.

### Stability of Candidate Reference Genes

#### Imidacloprid

**FIGURE 3:**
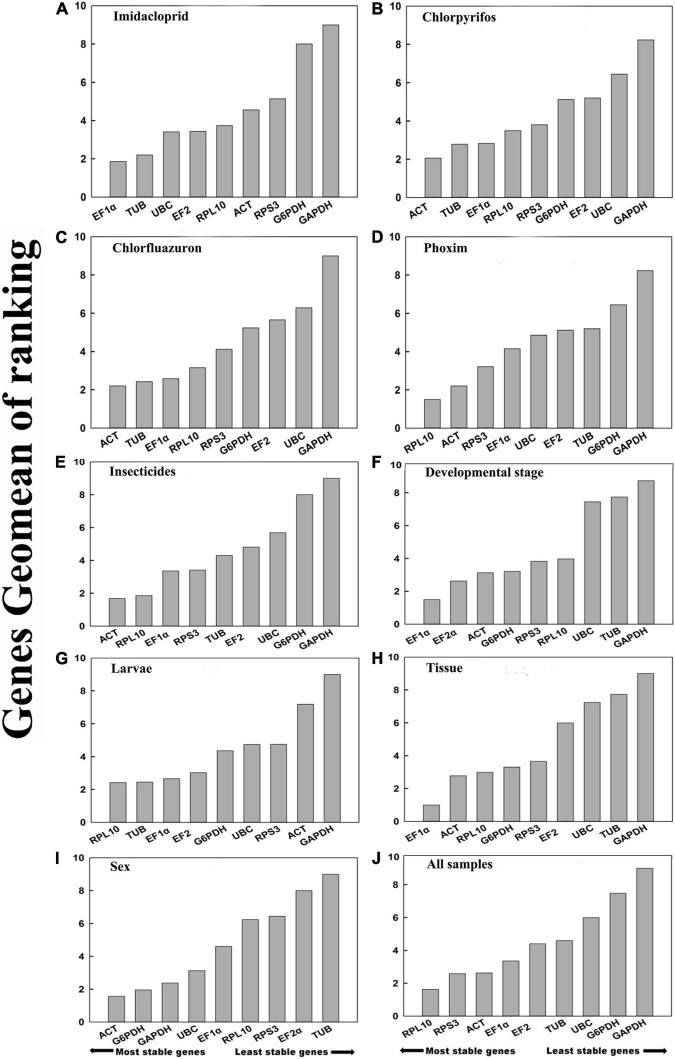
Stability of candidate reference genes in *B. odoriphaga* under various experimental conditions. In a RefFinder analysis, increasing Geomean values correspond to decreasing gene expression stability. The Geomean values for the following *B. odoriphaga* samples are presented: **(A)** imidacloprid: samples treated with imidacloprid; **(B)** chlorpyrifos: samples treated with chlorpyrifos; **(C)** chlorfluazuron: samples treated with chlorfluazuron; **(D)** phoxim:samples treated with phoxim; **(E)** insecticide treatment: adult samples treated with different insecticides; **(F)** developmental stage: samples for all developmental stages; **(G)** larvae: samples for larvae; **(H)** tissue: samples for different tissues; **(I)** adult samples for different sex; and **(J)** all samples: all samples for all treatments. The candidate reference genes are as follows: *EF-1*α, *elongation factor 1*α; *ACT*, *actin2*; *EF-2*α, *elongation factor 2*α; *G6PDH*, *glucose-6-phosphate dehydrogenase*; *GAPDH*, *glyceraldehyde-3-phosphate dehydrogenase*; *RPL10*, *ribosomal protein L10*; *RPS3*, *ribosomal protein S3*; *UBC*, *ubiquitin-conjugating enzyme*; *TUB*, *α-tubulin.*

**FIGURE 4:**
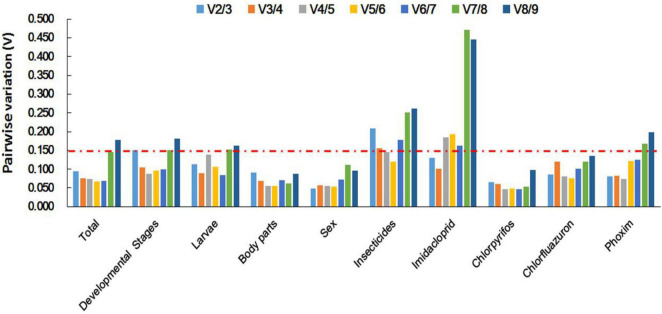
Optimal number of reference genes for accurate normalization as determined by geNorm. The Vn/*n* + 1 value indicates the pairwise variation (*Y*-axis) between two sequential normalization factors and was used to determine the optimal number of reference genes for an accurate data normalization. *A*-value < 0.15 indicates that an additional reference gene will not significantly improve the normalization.

Based on Δ*Ct* and the BestKeeper analyses, *TUB* and *EF1*α were the most stable genes (Table 2). However, the NormFinder analysis indicated *RPL10* and *ACT* as the most stable genes (Table 2). All four analyses revealed *G6PDH* and *GAPDH* as the least stable genes (Table 2). The rank order for gene stability in the imidacloprid determined using RefFinder was as follows (most to least stable): *EF1*α, *TUB*, *UBC*, *EF2*α, *RPL10*, *ACT*, *RPS3*, *G6PDH*, and *GAPDH* (Figure 3A). The geNorm data indicated that the pairwise variation value for V2/3 was less than the proposed 0.15 cut-off (Figure 4). The RefFinder analysis suggested that *EF1*α and *TUB* are required to normalize target gene expression levels under imidacloprid stress (Table 3).

**TABLE 2:** Expression stability of the nine candidate reference genes in *B. odoriphaga* under various experimental conditions.

Condition	Rank	Δ*Ct*		BestKeeper		NormFinder		geNorm	
		Gene Name	SV	Gene Name	SD	Gene Name	SV	Gene Name	SV
Imidacloprid	1	TUB	1.60	EF1α	0.32	RPL10	0.36	EF1α	0.25
	2	EF1α	1.70	TUB	0.38	ACT	0.40	EF2α	0.25
	3	UBC	1.72	UBC	0.41	TUB	1.05	UBC	0.38
	4	RPL10	1.73	EF2α	0.50	RPS3	1.07	TUB	0.40
	5	EF2α	1.74	RPS3	1.16	UBC	1.22	RPS3	0.61
	6	ACT	1.76	ACT	1.25	EF1α	1.30	ACT	0.82
	7	RPS3	1.80	RPL10	1.25	EF2α	1.40	RPL10	0.97
	8	G6PDH	3.42	G6PDH	2.82	G6PDH	3.18	G6PDH	1.64
	9	GAPDH	4.00	GAPDH	3.21	GAPDH	4.00	GAPDH	2.18
Chlorpyrifos	1	ACT	0.37	TUB	1.30	ACT	0.12	EF1α	0.15
	2	RPL10	0.39	EF1α	1.31	RPL10	0.18	TUB	0.16
	3	EF1α	0.40	EF2α	1.32	G6PDH	0.20	RPS3	0.20
	4	RPS3	0.41	RPS3	1.38	UBC	0.25	ACT	0.26
	5	TUB	0.41	RPL10	1.46	EF1α	0.26	RPL10	0.28
	6	G6PDH	0.44	ACT	1.53	RPS3	0.28	G6PDH	0.31
	7	UBC	0.48	G6PDH	1.60	TUB	0.30	UBC	0.34
	8	EF2α	0.58	UBC	1.68	EF2α	0.47	EF2α	0.37
	9	GAPDH	0.89	GAPDH	2.15	GAPDH	0.88	GAPDH	0.48
Chlorfluazuron	1	RPS3	061	TUB	0.27	ACT	0.18	RPS3	0.28
	2	RPL10	0.62	EF2α	0.50	EF1α	0.24	UBC	0.28
	3	UBC	0.65	EF1α	0.62	RPS3	0.24	RPL10	0.30
	4	ACT	0.68	ACT	1.01	UBC	0.25	ACT	0.39
	5	EF1α	0.69	UBC	1.03	RPL10	0.28	EF1α	0.41
	6	G6PDH	0.73	RPS3	1.08	G6PDH	0.49	G6PDH	0.47
	7	EF2α	0.77	RPL10	1.14	EF2α	0.60	EF2α	0.55
	8	GAPDH	1.15	G6PDH	1.23	GAPDH	1.07	GAPDH	0.63
	9	TUB	1.28	GAPDH	1.58	TUB	1.20	TUB	0.81
Phoxim	1	RPL10	1.33	TUB	0.60	RPL10	0.13	ACT	0.22
	2	ACT	1.35	EF2α	1.15	ACT	0.13	RPL10	0.23
	3	RPS3	1.44	EF1α	1.19	RPS3	0.15	RPS3	0.29
	4	UBC	1.50	RPS3	2.23	UBC	0.20	EF1α	0.32
	5	EF1α	1.52	RPL10	1.32	EF1α	0.35	UBC	0.38
	6	G6PDH	1.52	ACT	1.35	G6PDH	0.68	G6PDH	0.51
	7	EF2α	1.58	UBC	1.55	EF2α	0.78	EF2α	0.65
	8	GAPDH	2.20	G6PDH	1.88	GAPDH	1.52	GAPDH	0.84
	9	TUB	2.48	GAPDH	2.40	TUB	1.78	TUB	1.12
Insecticides	1	RPL10	1.12	TUB	0.68	ACT	0.20	ACT	0.55
	2	ACT	1.15	EF1α	1.08	RPL10	0.21	RPL10	0.55
	3	RPS3	1.18	EF2α	1.17	RPS3	0.52	RPS3	0.62
	4	EF1α	1.20	ACT	1.32	EF1α	0.69	EF1α	0.67
	5	EF2α	1.31	RPS3	1.32	UBC	0.90	UBC	0.71
	6	UBC	1.31	RPL10	1.50	EF2α	0.90	EF2α	0.74
	7	TUB	1.70	UBC	1.57	TUB	1.39	TUB	0.91
	8	G6PDH	1.90	G6PDH	1.84	G6PDH	1.58	G6PDH	1.21
	9	GAPDH	2.42	GAPDH	2.40	GAPDH	2.29	GAPDH	1.49
Developmental stages	1	EF1α	0.69	RPS3	0.38	EF1α	0.21	EF1α	0.35
	2	EF2α	0.70	RPL10	0.41	EF2α	0.24	ACT	0.36
	3	G6PDH	0.73	G6PDH	0.59	G6PDH	0.31	EF2α	0.38
	4	ACT	0.74	EF2α	0.61	ACT	0.44	G6PDH	0.51
	5	RPL10	0.79	EF1α	0.62	RPL10	0.55	RPL10	0.54
	6	RPS3	0.81	ACT	0.75	RPS3	0.56	RPS3	0.55
	7	UBC	0.99	TUB	0.93	UBC	0.79	UBC	0.69
	8	TUB	1.04	GAPDH	1.04	TUB	0.91	TUB	0.77
	9	GAPDH	1.19	UBC	1.10	GAPDH	1.12	GAPDH	0.90
Larvae	1	EF1α	0.62	RPL10	0.46	EF2α	0.26	RPL10	0.24
	2	EF2α	0.63	RPS3	0.49	EF1α	0.40	TUB	0.25
	3	TUB	0.64	TUB	0.53	UBC	0.45	G6PDH	046
	4	UBC	0.65	G6PDH	0.64	TUB	0.48	RPS3	0.48
	5	RPL10	0.70	EF1α	0.81	G6PDH	0.50	EF1α	0.52
	6	G6PDH	0.71	EF2α	0.83	ACT	0.54	UBC	0.58
	7	ACT	0.73	UBC	0.88	RPL10	0.55	EF2α	0.60
	8	RPS3	0.75	ACT	0.94	RPS3	0.55	ACT	0.63
	9	GAPDH	1.42	GAPDH	1.35	GAPDH	1.23	GAPDH	0.78
Tissues	1	EF1α	0.41	EF1α	0.33	EF1α	0.12	EF1α	0.15
	2	RPL10	0.41	ACT	0.39	RPL10	0.13	ACT	0.16
	3	RPS3	0.42	G6PDH	0.39	RPS3	0.15	G6PDH	0.21
	4	G6PDH	0.43	RPS3	0.48	ACT	0.20	RPL10	0.23
	5	ACT	0.45	RPL10	0.50	G6PDH	0.21	RPS3	0.27
	6	EF2α	0.50	EF2α	0.60	EF2α	0.32	EF2α	0.32
	7	UBC	0.61	UBC	0.64	UBC	0.48	TUB	0.41
	8	TUB	0.66	TUB	0.73	TUB	0.57	UBC	0.49
	9	GAPDH	0.87	GAPDH	0.81	GAPDH	0.79	GAPDH	0.55
Sex	1	ACT	0.45	G6PDH	0.13	G6PDH	0.14	ACT	0.15
	2	GAPDH	0.46	UBC	0.16	ACT	0.18	GAPDH	0.16
	3	G6PDH	0.48	ACT	0.17	UBC	0.23	EF1α	0.19
	4	UBC	0.50	GAPDH	0.21	GAPDH	0.25	UBC	0.20
	5	EF1α	0.51	RPS3	0.29	EF1α	0.38	G6PDH	0.25
	6	RPL10	0.57	EF1α	0.32	RPL10	0.47	RPL10	0.29
	7	RPS3	0.64	RPL10	0.38	RPS3	0.48	RPS3	0.36
	8	EF2α	0.85	EF2α	0.71	EF2α	0.75	EF2α	0.49
	9	TUB	0.92	TUB	0.82	TUB	0.87	TUB	0.62
All samples	1	RPL10	1.06	TUB	0.84	RPL10	0.32	RPL10	0.52
	2	ACT	1.07	EF1α	0.95	ACT	0.43	RPS3	0.53
	3	RPS3	1.11	EF2α	1.17	RPS3	0.51	ACT	0.64
	4	EF1α	1.13	ACT	1.21	EF1α	0.64	EF1α	0.65
	5	EF2α	1.22	RPS3	1.34	EF2α	0.71	EF2α	0.68
	6	UBC	1.23	UBC	1.40	UBC	0.87	UBC	0.70
	7	G6PDH	1.56	RPL10	1.42	G6PDH	1.23	TUB	0.92
	8	TUB	1.62	G6PDH	1.68	TUB	1.44	G6PDH	1.15
	9	GAPDH	2.05	GAPDH	2.15	GAPDH	1.98	GAPDH	1.34

**TABLE 3:** Most stable reference genes in *B. odoriphaga* under different experimental conditions.

Experimental conditions	Reference genes (most stable)	Experimental conditions	Reference genes (most stable)
Imidacloprid	*EF1α, TUB*	Developmental stage	*EF1*α, *EF2*α
Chlorpyrifos	*ACT*, *TUB*	Larvae	*RPL10, TUB*
Chlorfluazuron	*ACT*, *TUB*	Tissue	*EF1α, ACT*
Phoxim	*RPL10, ACT*	Sex	*ACT*, *G6PDH*
Insecticides	*ACT*, *RPL10*, *EF1*α	All samples	*RPL10, RPS3*

#### Chlorpyrifos

Both Δ*Ct* and NormFinder identified *ACT* and *RPL10* as the most stable genes across chlorpyrifos samples (Table 2). In contrast, BestKeeper and geNorm detected *TUB* and *EF1*α as the most stable genes (Table 2). All analyses indicated that *EF2*α and *GAPDH* were the least stable genes. The RefFinder results for chlorpyrifos indicated the rank order for gene stability was as follows (most to least stable): *ACT*, *TUB*, *EF1*α, *RPL10*, *RPS3*, *G6PDH*, *EF2*α, *UBC*, and *GAPDH* (Figure 3B). The geNorm data indicated that all pairwise values were less than the proposed 0.15 cut-off (Figure 4). Based on the RefFinder analysis, *ACT* and *TUB* are required to normalize target gene expression levels across chlorpyrifos (Table 3).

#### Chlorfluazuron

The Δ*Ct* analyses identified *RPS3*, *RPL10*, and *UBC* as the most stable genes across chlorfluazuron samples (Table 2). Similar results were obtained by geNorm (Table 2). However, BestKeeper identified *TUB* as the most stable gene (Table 2). The least stable gene was identified as *TUB* according to Δ*Ct*, NormFinder, and geNorm. The RefFinder data indicated that the rank order for gene stability among chlorfluazuron samples was as follows (most to least stable): *ACT*, *TUB*, *EF1*α, *RPL10*, *RPS3*, *G6PDH*, *EF2*α, *UBC*, and *GAPDH* (Figure 3C). The geNorm analysis revealed that all pairwise variation values were less than the proposed 0.15 cut-off (Figure 4). The RefFinder analysis suggested that *ACT* and *TUB* are required to normalize target gene expression levels in chlorfluazuron-treated *B. odoriphaga* (Table 3).

#### Phoxim

All analyses except the BestKeeper indicated that *RPL10*, *ACT*, and *RPS3* were the most stable genes, while *GAPDH* and *TUB* were the least stable genes (Table 2). In contrast, BestKeeper identified *TUB* as the most stable gene (Table 2). The rank order for gene stability determined using RefFinder was as follows (most to least stable): *RPL10*, *ACT*, *RPS3*, *EF1*α, *UBC*, *EF2*α, *TUB*, *G6PDH*, and *GAPDH* (Figure 3D). The geNorm data indicated that the pairwise variation value for V2/3 was less than the proposed 0.15 cut-off (Figure 4). The RefFinder analysis showed that target gene expression levels under phoxim stress conditions should be normalized against the expression of *RPL10* and *ACT* (Table 3).

#### Integrative Analysis of Reference Genes Under Insecticides’ Stress

Regarding the insecticides’ stress effects, the Δ*Ct*, geNorm, and NormFinder analyses indicated that the most stable genes were *RPL10*, *ACT*, and *RPS3*, whereas BestKeeper identified *TUB*, *EF1*α, and *EF2*α as the most stable genes (Table 2). All four analyses identified *GAPDH* and *G6PDH* as the least stable genes (Table 2). The RefFinder data indicated the rank order for gene stability was as follows (most to least stable): *ACT*, *RPL10*, *EF1*α, *RPS3*, *TUB*, *EF2*α, *UBC*, *G6PDH*, and *GAPDH* (Figure 3E). The geNorm analysis revealed that the pairwise variation value of V4/5 was less than the proposed 0.15 cut-off (Figure 4). The RefFinder analysis suggested that *EF1*α, *TUB*, and *UBC* are required to normalize target gene expression levels in *B. odoriphaga* under insecticides stress (Table 3).

#### Developmental Stages

Regarding the analyzed developmental stages, the Δ*Ct* method, NormFinder, and geNorm, but not BestKeeper, indicated that *EF-1*α was the most stable gene and *GAPDH* and *TUB* were the least stable genes (Table 2). The BestKeeper analysis identified *RPS3* and *RPL10* as the most stable genes. In contrast, *UBC* was the least stable gene. The RefFinder analysis indicated the rank order for reference gene stability as follows (most to least stable): *EF1*α, *EF2*α, *ACT*, *G6PDH*, *RPS3*, *RPL10*, *UBC*, *TUB*, and *GAPDH* (Figure 3F). The geNorm analysis revealed that all pairwise variation values were less than the proposed 0.15 cut-off, except for V8/9 (Figure 4). A value less than 0.15 indicates that adding another reference gene will not change the normalization. The RefFinder analysis revealed that *EF1*α and *EF2*α are required for normalizing target gene expression levels in different *B. odoriphaga* developmental stages (Table 3).

#### Larvae

Both Δ*Ct* and NormFinder identified *EF1*α and *EF2*α as the most stable genes and *RPL10* as a moderately stable gene in larval samples (Table 2). However, the BestKeeper and geNorm analysis identified *RPL10* as the most stable gene. According to the four algorithms, *GAPDH* was considered the least stable gene (Table 2). The rank order for gene stability based on the RefFinder results was as follows (most to least stable): *RPL10*, *TUB*, *EF1*α, *EF2*α, *G6PDH*, *UBC*, *RPS3*, *ACT*, and *GAPDH* (Figure 3G). The geNorm analysis indicated that the pairwise value of V2/3 was less than the proposed 0.15 cut-off (Figure 4). The RefFinder analysis suggested that *RPL10* and *TUB* are required to normalize target gene expression levels in *B. odoriphaga* larval samples (Table 3).

#### Tissues

According to the four algorithms, the most stable gene was *EF1*α, and the least stable genes were *GAPDH*, *UBC*, and *TUB* across the tissues (Table 2). According to RefFinder, the reference gene stability rank order across tissues was as follows (most to least stable): *EF1*α, *ACT*, *RPL10*, *G6PDH*, *RPS3*, *EF2*α, *UBC*, *TUB*, and *GAPDH* (Figure 3H). The geNorm analysis results showed that all pair-wise variation values were less than the proposed 0.15 cut-off. The RefFinder analysis indicated *EF1*α and *ACT* are required for normalizing target gene expression levels in different *B. odoriphaga* tissues (Table 3).

#### Sex

Both Δ*Ct* and geNorm identified *ACT* and *GAPDH* as the most stable genes across sex samples (Table 2). The BestKeeper and NormFinder analysis also identified *G6PDH* as the most stable gene, while *UBC* and *ACT* were the second and third stable genes, respectively (Table 2). According to the four algorithms, *EF2*α and *TUB* were identified as the least stable genes (Table 2). The rank order for gene stability among the examined sex samples based on the RefFinder results was as follows (most to least stable): *ACT*, *G6PDH*, *GAPDH*, *UBC*, *EF1*α, *RPL10*, *RPS3*, *EF2*α, and *TUB* (Figure 3I). The geNorm analysis indicated that the pairwise value of V2/3 was less than the proposed 0.15 cut-off (Figure 4). The RefFinder analysis suggested that *ACT* and *G6PDH* are required to normalize target gene expression levels in *B. odoriphaga* sex samples (Table 3).

#### Overall Ranking of *Bradysia odoriphaga* Reference Genes

Based on the RefFinder analysis, the overall rank order for the stability of *B. odoriphaga* genes was as follows (most to least stable): *RPL10*, *RPS3*, *ACT*, *EF1*α, *EF2*α, *TUB*, *UBC*, *G6PDH*, and *GAPDH* (Figure 3J). The geNorm analysis indicated that all pairwise variation values were less than the proposed 0.15 cut-off, except for V8/9 (Figure 4). The RefFinder data suggested that *RPL10* and *RPS3* are suitable internal reference genes for normalizing target gene expression levels in *B. odoriphaga* (Table 3).

## Discussion

It is unquestionably true that gene expression quantification has never been easier than it is now, thanks to RT-qPCR technology. However, extreme care must be taken to avoid erroneous results (Liang et al., 2014). One of the most common strategies for correcting experimental errors introduced during the steps of RT-qPCR analysis is the normalization of RT-qPCR data with reference genes (Pinheiro et al., 2020). Inappropriate reference gene selection can obscure or magnify real biological changes caused by changes in reference gene expression (Zhu et al., 2014). Therefore, a reference gene with low expression variation must be chosen to ensure accurate normalization and avoid inaccurate quantification of gene expression (Huggett et al., 2005).

Earlier studies on reference genes evaluation and validation in insects under insecticide stress reported that the expression of reference genes varies under different insecticide stress even if they belong to the same group of insecticides (Liang et al., 2014). These findings further demonstrate that there is no single universal reference available under different conditions. Therefore, identifying suitable reference genes is critical for obtaining a reliable estimate for gene expression levels under different conditions.

The present study evaluated the expression stability of nine candidate reference genes in *B. odoriphaga* under four insecticides commonly applied for controlling this pest. Moreover, the stability of these selected candidate genes was also assessed in developmental stages, sexes, and different tissues of *B. odoriphaga*.

The assessment of RNA integrity and amplification efficiency must be conducted prior to RT-qPCR based analysis of genes expression. In the present work, RNA integrity results showed that the OD ratio (A260/A280) of all RNA samples varied between 1.8 and 2.0, and the amplification efficiency of the nine candidates ranged from 90 to 110% (*R*^2^ > 0.996) (Table 1). Thus, RNA quality and amplification were of sufficient quality to be used in RT-qPCR. Our RNA quality and amplification results agree with other reference gene validation studies conducted on the other insects (Shakeel et al., 2015; Pinheiro et al., 2020).

Our results of reference genes expression stability offered by five algorithms (geNorm, NormFinder, BestKeeper, Delta Ct, and RefFinder) indicated that the ranking order was different, such as *TUB* and *EF1*α were ranked as the most stable reference genes by Δ*Ct*. In contrast, NormFinder ranked *RPL10* and *ACT* as the most stable reference genes under imidacloprid stress. Similarly, geNorm indicated *RPS3* and *UBC* as the most stable reference genes, whereas BestKeeper ranked *TUB* and *EF2*α as the most stable reference genes under chlorfluazuron stress. These discrepancies in the ranking order by different algorithms within the same tested insecticide might be because of the various analytical methods used (Shakeel et al., 2018). On the other hand, the difference in ranking of the reference genes under the stress of different insecticides in this study demonstrates the importance of evaluating their use under different sets of insecticides. Our findings provide more comprehensive information regarding reference genes selection under insecticide stress compared to the previous studies on *B. odoriphaga* (Shi et al., 2016; Tang et al., 2019).

The *ACT* gene, which is most frequently used as a reference gene, encodes a major structural protein that maintains organisms’ life activity and exhibits conservative structure during evolution. In the present study, our results demonstrated that *ACT* expression was highly stable under insecticide stress (chlorpyrifos, chlorfluazuron) and other experimental conditions, including tissues and both sexes, and developmental stages. Coincidentally, the results are consistent with the earlier reports. For example, *ACT* was identified as one of the most stable reference genes for normalizing target gene expression in *Spodoptera litura* treated with insecticides (Lu et al., 2013). Additionally, *ACT* expression was revealed to be most stable in *Locusta migratoria* under different insecticides stress (Yang et al., 2014). *ACT* also showed high stability in other insects under different experimental sets, such as in *Plutella xylostella* and *Chilo suppressalis* under different development stages (Teng et al., 2012), *D. melanogaster* after heat-stress (Ponton et al., 2011), *Schistocerca gregaria* in fifth instar nymphs (Van Hiel et al., 2009), and *Orchesella cincta* overall treatments (de Boer et al., 2009). Quite the contrary, *ACT* was a less stable reference gene for gene expression analyses in *Bombyx mori*, *Spodoptera exigua* (Teng et al., 2012), *Coleomegilla maculata* (Yang C. et al., 2015), *Coccinella septempunctata* (Yang et al., 2016), and *Hippodamia convergens* (Pan et al., 2015). In this study, *ACT* was not an ideal reference gene for the larval stage in *B. odoriphaga*. Thus, there is no single universal reference gene suitable for all insects and under all conditions, even the most commonly used housekeeping gene responds differently to various experimental conditions.

The *TUB* gene is assigned to the Eukaryotic structural gene family, and encodes cytoskeletal structure proteins that involve in the regulation of cell division, shape, motility, and intracellular activity. In previous studies, *TUB* exhibited a stable expression, for example, *Nilaparvata lugens* for geographic population (Yuan et al., 2014), *Sogatella furcifera* at different developmental stages and under different temperature stress (An et al., 2016), *Thitarodes armoricanus* for the fungal infections (Liu et al., 2016), and *Bemisia tabaci* MED across all sample sets (Dai et al., 2017). In this study, the stability of *TUB* was variable under different treatments in *B. odoriphaga*. It exhibited a stable expression under chlorpyrifos, imidacloprid, and chlorfluazuron stress, whereas its expression was unstable across different developmental stages and tissues. Similar results have also been noted in *C. maculata* (Yang C. et al., 2015). The above results clearly suggest that determining candidates and evaluating their suitability is required for each experimental condition.

In the present study, the *EF1*α gene expression levels was stable across different developmental stages, tissues, and under the treatment of imidacloprid. Indeed, *EF1*α has been commonly picked as reference genes across different developmental stages and temperature in many other insect species, such as *Sesamia inferens* (Sun et al., 2015), *L. migratoria* (Yang et al., 2014), *Frankliniella occidentalis* (Zheng et al., 2014), and *H. convergens* (Pan et al., 2015). However, *EF1*α was considered unstable in developmental stages and tissues, again, in *B. odoriphaga* (Shi et al., 2016). This discrepancy between our study and previous study might be caused by different candidate reference genes, diet, population, temperature, and photoperiods.

Notably, the *GAPDH* gene, which encodes a key enzyme involved in the energy metabolism and ranked as the fourth most widely used reference gene, showed poor stability among almost all experimental conditions in this study. There are also some reports suggesting that the *GAPDH* was not suitable to be used as reference gene under the specific condition in some species, for example, *Bactrocera dorsalis* in difference tissues (Shen et al., 2010), *Musca domestica* (Zheng et al., 2014), and *Lucilia cuprina* (Bagnall and Kotze, 2010) in difference developmental stages. On the other hand, *GADPH* was used as the most stable reference gene, such as *S. litura* in developmental stage and under temperature stress (Lu et al., 2013), *P. xylostella* in mechanical injury (Fu et al., 2013), *Euscelidius variegatus*, and *Macrosteles quadripunctulatus* by phytoplasma infection (Galetto et al., 2013). The results showed that the expression of candidate reference genes was not stable in all the tested conditions. Thus it is necessary to select different genes to normalize expression under different experimental conditions.

## Conclusion

In summary, there was no single universal reference gene that could be used in all situations. It is indispensable to validate the expression of candidate genes before using them as the internal controls in qPCR. A suite of reference genes was specifically recommended for each experimental condition in this study. The suitable reference genes in different experimental conditions were *EF1*α and *EF2*α in development stages; *EF1*α and *ACT* in tissues; *ACT* and *G6PDH* in sex; *RPL10* and *ACT* in phoxim treatment; *ACT* and *TUB* in chlorpyrifos treatment; *EF1*α and *TUB* in imidacloprid treatment; and *ACT* and *TUB* in chlorfluazuron treatment. The results of our experiment can be used for the further studies in *B. odoriphaga*.

## Data Availability Statement

The original contributions presented in the study are included in the article/supplementary material, further inquiries can be directed to the corresponding author.

## Ethics Statement

The animal study was reviewed and approved by the Animal Ethics Committee of Heilongjiang University. Written informed consent was obtained from the owners for the participation of their animals in this study.

## Author Contributions

FY and HF conceived and designed the research. TH, ZX, ChaoL, and CY conducted the experiments. ChunL and TW analyzed the data. HF wrote the manuscript. FS, FF, and FY revised the manuscript. All authors have read and approved the manuscript.

## Conflict of Interest

The authors declare that the research was conducted in the absence of any commercial or financial relationships that could be construed as a potential conflict of interest.

## Publisher’s Note

All claims expressed in this article are solely those of the authors and do not necessarily represent those of their affiliated organizations, or those of the publisher, the editors and the reviewers. Any product that may be evaluated in this article, or claim that may be made by its manufacturer, is not guaranteed or endorsed by the publisher.

## Footnotes

1
http://www.ncbi.nlm.nih.gov/tools/primer-blast/index.cgi?LINK_LOC=BlastHome

2
http://mfold.rna.albany.edu/?q=mfold/DNA-Folding-Form

3 http://www.mdl.dk/publications NormFinder.htm

4
http://GeNorm.cmgg.be

5
http://www.wzw.tum.de/gene-quantification/bestkeeper.html

6
https://www.heartcure.com.au/for-researchers
